# Neuroprotective Effects of Fluoxetine Derivative 4-[3-Oxo-3-(2-trifluoromethyl-phenyl)-propyl]-morpholinium Chloride (OTPM) as a Potent Modulator of Motor Deficits and Neuroinflammatory Pathways in LPS-Induced BV-2 Microglial Cells and MPTP-Induced Parkinsonian Models

**DOI:** 10.3390/ph18121799

**Published:** 2025-11-26

**Authors:** Seong-Mook Kang, Rengasamy Balakrishnan, Hyun Myung Ko, Ju-Young Park, Hemant Kumar, Byungwook Kim, Sung-Hwa Yoon, Dong-Kug Choi

**Affiliations:** 1 Department of Applied Life Sciences, BK21 Program, Graduate School, Konkuk University, Chungju 27478, Republic of Korea; tjdanranr@gmail.com (S.-M.K.); kohm@woosuk.ac.kr (H.M.K.); 2 Department of Biotechnology, Research Institute of Inflammatory Disease (RID), College of Biomedical and Health Science, Konkuk University, Chungju 27478, Republic of Korea; balakonkuk@kku.ac.kr; 3 Molecular Science and Technology Research Center, Ajou University, Suwon 16499, Republic of Korea; pink1209@ajou.ac.kr; 4 Department of Pharmacology and Toxicology, National Institute of Pharmaceutical Education and Research (NIPER), Ahmedabad 382355, India; hemant@niperahm.res.in; 5 Department of Medical and Molecular Genetics, Indiana University School of Medicine, Indianapolis, IN 46202, USA; bk18@iu.edu; 6 Department of Molecular Science and Technology, Ajou University, Suwon 16499, Republic of Korea; gator503@naver.com

**Keywords:** Parkinson’s disease, neuroprotection, OTPM, MPTP, microglial activation, neuroinflammation

## Abstract

**Background/Objectives:** Parkinson’s disease (PD) is the second most common neurodegenerative disease (NDD), marked by the progressive loss of dopaminergic neurons in the substantia nigra that causes motor dysfunction. Growing evidence indicates that neuroinflammation plays a crucial role in the onset and progression of PD, though the exact mechanisms are still unclear. In this study, we examined the anti-inflammatory and neuroprotective effects of 4-[3-oxo-3-(2-trifluoromethyl-phenyl)-propyl]-morpholinium chloride (OTPM), a fluoxetine derivative and selective serotonin reuptake inhibitor, in both lipopolysaccharide (LPS)-stimulated BV-2 microglial cells and an MPTP-induced mouse model of PD. **Methods:** C57BL/6 mice were orally administered OTPM (10 mg/kg b.w.) for 7 days and intraperitoneally injected with MPTP (20 mg/kg b.w.) for one day, with four injections at 2 h intervals. Bradykinesia was assessed using the Y-maze and Pole tests. Protein and mRNA levels were examined in vitro and in vivo using Western blotting and RT-PCR. Immunofluorescence was used to assess microglial and astrocyte activation. **Results:** In vitro, OTPM significantly decreased nitric oxide (NO) production (*p* < 0.001) and suppressed the protein and mRNA expression of iNOS (*p* < 0.001), COX-2 (*p* < 0.001), and pro-inflammatory cytokines, including IL-β (*p* < 0.001), IL-6 (*p* < 0.001), and TNF-α (*p* < 0.01), in LPS-activated BV-2 microglia. Further mechanistic studies showed that OTPM inhibited NF-κB phosphorylation and blocked its nuclear translocation, thereby reducing inflammatory signaling. In vivo, treatment with OTPM (10 mg/kg for 7 days) significantly reduced the MPTP-induced activation of microglia (MAC-1) and astroglia (GFAP) in the brain and improved behavioral deficits associated with PD, as assessed in the Y-maze and pole tests. **Conclusions:** Overall, these results reveal that OTPM has strong anti-neuroinflammatory and neuroprotective properties, suggesting its potential as a new therapeutic candidate for PD and other disorders associated with neuroinflammation.

## 1. Introduction

Parkinson’s disease (PD) is one of the major neurodegenerative diseases (NDDs) associated with the advancement of age, which affects motor function. Clinical features such as rigidity, tremors, and bradykinesia, along with slurred speech, impaired gait, and cognitive dysfunction, occur due to disturbed motor function [1,2]. Dysfunction or loss of dopaminergic neurons in the substantia nigra of the midbrain, along with Lewy body formation and cytoplasmic aggregation of α-synuclein, plays a role in the pathogenesis of PD [3]. Although the exact mechanisms of neuronal degeneration are not fully understood, factors such as lipid peroxidation, oxidative stress, mitochondrial dysfunction, neuroinflammation, and excitotoxicity are thought to contribute [4,5]. Among the multiple molecular mechanisms involved in PD, neuroinflammation is a key factor contributing to its development and progression. This inflammatory response is primarily mediated by glial cells in the central nervous system (CNS), particularly microglia and astrocytes [6]. However, under pathological conditions, microglia release pro-inflammatory mediators, including inducible nitric oxide synthase (iNOS), cyclooxygenase-2 COX-2, interleukin-6 (IL-6), interleukin-1β (IL-1β), and tumor necrosis factor-alpha (TNF-α), which can subsequently cause neurotoxic effects, promote neuronal injury, and lower dopamine levels in the brain. Excessive neuroinflammation contributes to the progression of numerous NDDs [7]. Therefore, controlling microglia-driven neuroinflammatory responses is crucial. Current treatments, like dopamine replacement and related drugs, relieve symptoms but do not stop disease progression. Additionally, prolonged use of these therapies often causes serious side effects and motor complications, such as dyskinesia [8]. Consequently, there is an urgent need to develop safer and more effective alternative treatments.

MPTP is a commonly used neurotoxin showing similar clinical and neuropathological findings to those observed in PD patients; these observations led to the establishment of MPTP as a common neurotoxin to induce Parkinsonism in several animal models [9,10]. Previous studies have shown that systemic administration of MPTP rapidly induces neuroinflammatory responses in the brain. These responses involve activation of microglia and astrocytes, which are triggered by impaired dopaminergic neuron function and contribute to neuroinflammatory alterations [11,12]. LPS can stimulate various neuroinflammatory signaling pathways, particularly NF-κB signaling, an inducible transcription factor that is more active in the CNS than in the periphery and plays a significant role in PD-related neuroinflammation [13]. In addition, NF-κB is known to be involved in learning and memory, as well as neuronal degeneration in PD [14]. Therefore, inhibiting this pathway is a promising therapeutic strategy to reduce neuroinflammation and its associated neuronal damage in PD [15,16].

Fluoxetine, a selective serotonin reuptake inhibitor commonly used to treat depression and other disorders, has been shown to have antioxidant properties by reversing oxidative damage. Previous studies have shown that fluoxetine improves cognitive function in PD mice by modulating neuroinflammatory and neuronal apoptotic pathways, supporting its neuroprotective role in PD models [17,18]. Other reports have also emphasized fluoxetine’s ability to counteract neuroinflammation, suggesting its promise as a PD treatment [18,19]. In our ongoing search for potent neuroprotective and anti-inflammatory fluoxetine derivatives and analogs, OTPM showed significantly stronger anti-inflammatory activity than other synthesized analogs, effectively reducing inflammatory responses in microglial cells. Our recent findings further revealed that OTPM and its structurally related analog suppress NO production and LPS-induced inflammatory responses in microglial cells [20]. However, further studies are required to elucidate the precise molecular mechanisms through which fluoxetine and its analog OTPM confer neuroprotection in PD. Therefore, the present study aims to investigate the molecular mechanisms underlying OTPM’s effects, providing new insights into its neuroprotective and anti-neuroinflammatory roles in PD pathogenesis.

## 2. Results

### 2.1. OTPM Synthesis

**Figure 1:**
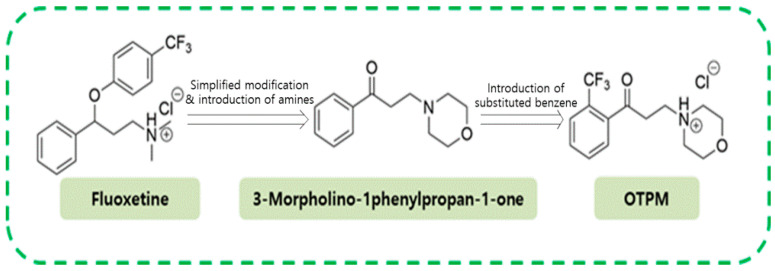
The structure of fluoxetine, a simplified morpholine analog of fluoxetine and OTPM.

As part of our program to find a potent inhibitor of NO production, we discovered that 3-morpholino-1-phenylpropan-1-one, a simplified morpholine analog of fluoxetine, inhibits NO production in LPS-stimulated BV-2 microglial cells without causing toxicity [21]. To improve the potency, a series of simplified morpholine analogs of fluoxetine were synthesized, and their inhibitory effects on NO production in LPS-induced BV-2 microglial cells were tested. Among the synthesized analogs, (4-(3-oxo-3-(2-(trifluoromethyl)phenyl)propyl)morpholin-4-ium chloride) (OTPM), possessing an ortho-positioned trifluoromethyl group in benzene, showed a significantly higher inhibitory activity on NO production and inhibited iNOS expression in LPS-induced BV-2 microglial cells. Therefore, we chose OTPM for further in vitro and in vivo experiments (Figure 1).

### 2.2. OTPM Inhibits NO Release Without Affecting the Viability of LPS-Treated BV-2 Microglial Cells

**Figure 2:**
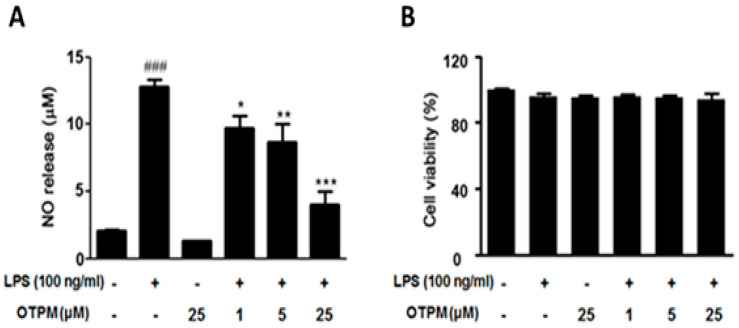
The effects of OTPM on nitric oxide (NO) production and cell viability in LPS-stimulated BV-2 microglia. BV-2 microglial cells were treated with 1, 5, or 25 μM OTPM for 1 h, then stimulated with 100 ng/mL LPS for 24 h. NO levels in the culture media were measured using the Griess reaction (**A**). Cell viability was assessed via the MTT assay (n = 6) (**B**). Data are expressed as mean ± SD from three independent experiments. Statistical significance: ^###^ *p* < 0.001 vs. control; * *p* < 0.05, ** *p* < 0.01, *** *p* < 0.001 vs. LPS treatment.

BV-2 microglial cells were pre-treated with OTPM at concentrations of 1, 5, and 25 µM for 1 h, followed by LPS (100 ng/mL) treatment for 24 h to evaluate its effect on LPS-induced NO production. LPS significantly increased NO levels (12.79 ± 1.13 µM) compared to control cells (2.07 ± 0.18 µM), whereas OTPM alone did not affect NO production (1.28 ± 0.07 µM). Pre-treatment with OTPM reduced NO production in a dose-dependent manner to 9.74 ± 1.26, 8.68 ± 1.91, and 4.02 ± 1.32 µM at 1, 5, and 25 µM, respectively (Figure 2A). Cell viability was assessed using the MTT assay, which measures mitochondrial activity of living cells. BV-2 cells treated with OTPM (1–25 µM), alone or in combination with LPS, showed no cytotoxicity, with control cell viability set at 100% (Figure 2B). These results indicate that OTPM effectively inhibits LPS-induced NO production without affecting cell viability in BV-2 microglial cells.

### 2.3. OTPM Attenuates iNOS and COX-2 Production in LPS-Stimulated BV-2 Microglial Cells

**Figure 3:**
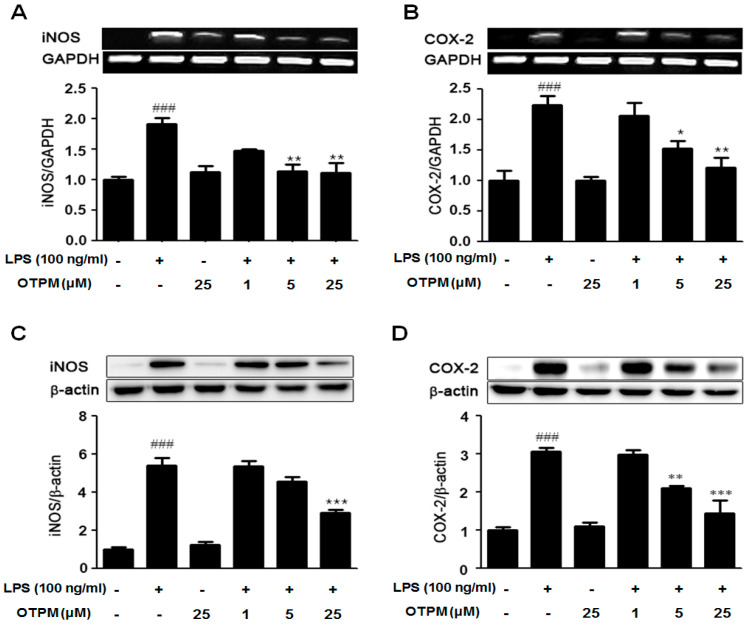
The effect of OTPM on iNOS and COX-2 expression in LPS-stimulated BV-2 microglial cells. BV-2 microglial cells were pretreated with OTPM at 1, 5, or 25 μM for 1 h, then stimulated with LPS (100 ng/mL) for 18 h. Total RNA was extracted for RT-PCR analysis of iNOS and COX-2 gene expression, with results shown as ratios to GAPDH (**A**,**B**). Protein lysates were analyzed via Western blot using anti-iNOS and anti-COX-2 antibodies, with results expressed as ratios to β-actin (**C**,**D**). GAPDH and β-actin served as internal controls for RT-PCR and Western blot analysis, respectively. Data are presented as mean ± SD from three independent experiments (n = 3). Statistical significance compared to the control group is indicated by ^###^ *p* < 0.001, and compared to the LPS-treated group by * *p* < 0.05, ** *p* < 0.01, or *** *p* < 0.001, determined by one-way ANOVA.

We next examined whether OTPM suppresses LPS-induced iNOS and COX-2 expression in BV-2 microglia. Cells were pretreated with OTPM (1, 5, and 25 µM) for 1 h, followed by LPS (100 ng/mL) stimulation for 18 h. LPS markedly increased iNOS and COX-2 expression at both mRNA and protein levels compared to controls. Pretreatment with OTPM significantly reduced iNOS and COX-2 expression at the mRNA (Figure 3A,B) and protein (Figure 3C,D) levels relative to the LPS-only group, indicating that OTPM effectively inhibits the production of these inflammatory mediators.

### 2.4. OTPM Attenuates the Production of Pro-Inflammatory Cytokines in LPS-Stimulated BV-2 Microglial Cells

**Figure 4:**
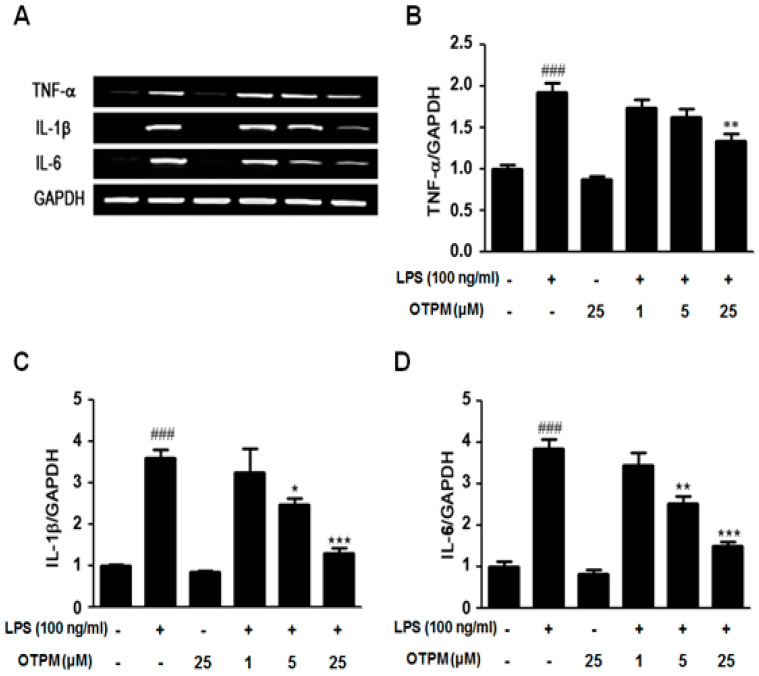
The effect of OTPM on pro-inflammatory cytokines in LPS-stimulated BV-2 microglial cells. Cells were pretreated with OTPM at concentrations of 1, 5, or 25 µM for 1 h prior to exposure to LPS (100 ng/mL). After a 6 h incubation with LPS, mRNA levels of TNF-α, IL-1β, and IL-6 were measured via RT-PCR (**A**). GAPDH served as an internal control. Results are presented as the ratios of TNF-α/GAPDH (**B**), IL-1β/GAPDH (**C**), and IL-6/GAPDH (**D**) across three independent experiments. Data are expressed as mean ± SD (n = 3). ^###^ *p* < 0.001, vs. control group; * *p* < 0.05, ** *p* < 0.01, *** *p* < 0.001, vs. LPS-treated group, determined by one-way ANOVA.

Pro-inflammatory cytokines are key mediators of microglia-driven inflammation. To assess the effects of OTPM, BV-2 microglial cells were treated with OTPM (1, 5, and 25 μM) in the presence or absence of LPS (100 ng/mL). Under basal conditions, TNF-α, IL-1β, and IL-6 were undetectable, but LPS treatment significantly increased their expression. Pretreatment with OTPM markedly inhibited LPS-induced production of TNF-α, IL-1β, and IL-6 (Figure 4A). Quantitative analysis confirmed that OTPM significantly reduced LPS-stimulated mRNA levels of TNF-α, IL-1β, and IL-6 (Figure 4B–D), indicating that OTPM effectively suppresses the production of these pro-inflammatory cytokines.

### 2.5. OTPM Inhibits LPS-Induced NF-κB Activation in BV-2 Microglial Cells

**Figure 5:**
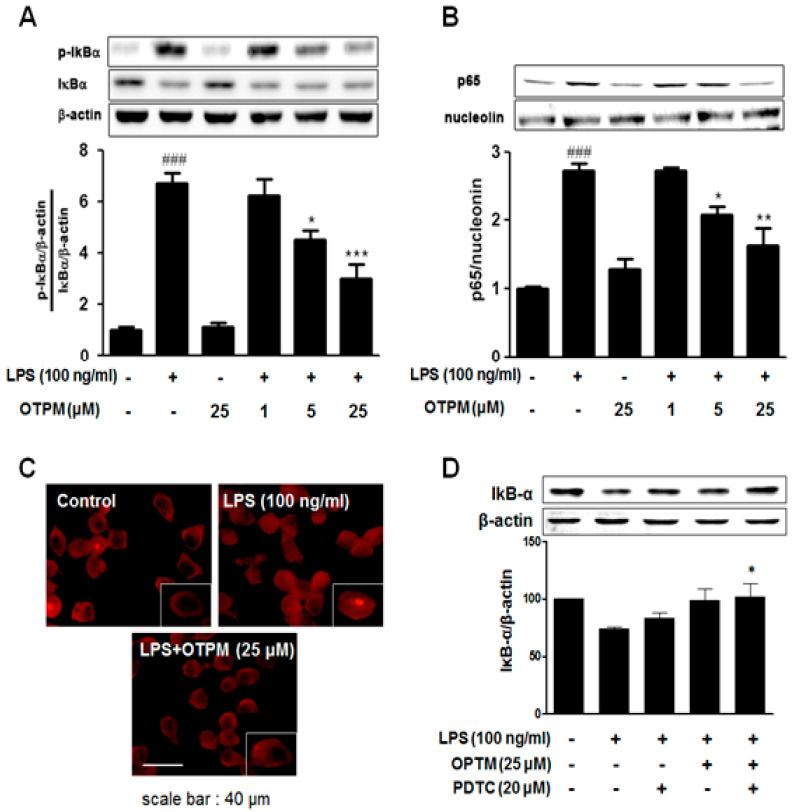
The effect of OTPM on NF-κB activation and IκB-α degradation in LPS-stimulated BV-2 microglial cells. The cells were treated with the indicated doses (1, 5, and 25 µM) of OTPM for 1 h before LPS treatment for Western blot analysis. Total nuclear protein was subjected to 10% SDS-PAGE followed by Western blot using IκB-α (**A**) and anti-NF-κB p65 (**B**). For immunofluorescence, cells were seeded at 5 × 10^4^ cells per well on 12 mm coverslips in 12-well plates. The cells were stimulated with 100 ng/mL LPS, either in the absence or presence of OTPM (25 µM), which was added 1 h before stimulation. Thirty minutes after adding the LPS, the subcellular location of the NF-κB p65 subunit was determined by immunofluorescence analysis (**C**). The combined effects of co-treatment (OTPM; 25 µM and PDTC; 20 µM) for 1 h before LPS treatment were analyzed by Western blot (**D**). Nucleolin and β-actin served as internal controls for Western blot, and data are presented as mean ± SD from three independent experiments (n = 3). Values are mean ± SD; ^###^ *p* < 0.001 vs. control group; * *p* < 0.05, ** *p* < 0.01, and *** *p* < 0.001 vs. LPS-treated group.

To investigate the mechanism of OTPM, NF-κB (p65) activation was examined in LPS-stimulated BV-2 microglial cells. Cells were pretreated with OTPM for 30 min before LPS stimulation, and NF-κB activity was assessed by the nuclear translocation of the p65 subunit (Figure 5B). Immunocytochemistry revealed that OTPM attenuated LPS-induced p65 nuclear translocation (Figure 5C). Additionally, OTPM significantly inhibited IκB-α phosphorylation (Figure 5A). This inhibitory effect on IκB-α phosphorylation was further enhanced when OTPM was combined with the NF-κB inhibitor PDTC in LPS-stimulated cells (Figure 5D). These findings suggest that OTPM suppresses the production of NO, iNOS, COX-2, and pro-inflammatory cytokines (TNF-α, IL-1β, and IL-6) by regulating NF-κB activation and IκB-α degradation in LPS-stimulated BV-2 microglial cells.

### 2.6. OTPM Attenuates Glial Activation in the MPTP-Induced PD Mouse Model

**Figure 6:**
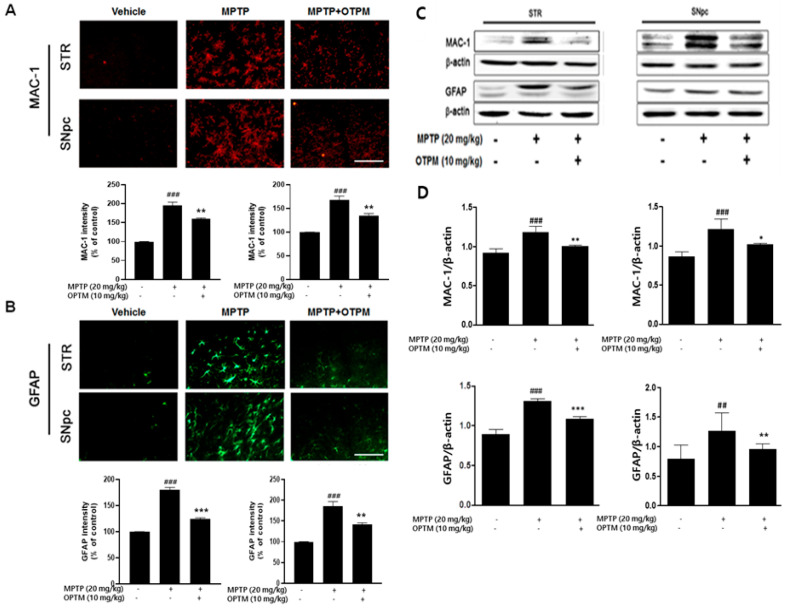
The effect of OTPM on MPTP-induced activation of microglial/astrocyte in PD mice. C57BL/6 mice were given OTPM at 10 mg/kg orally for 7 days before MPTP administration (20 mg/kg, intraperitoneally, 4 injections per day at 2 h intervals). Representative GFAP and MAC-1immunofluorescence staining were performed in the SNpc and STR, with MAC-1 (**A**) and GFAP (**B**) (n = 3). MAC-1 and GFAP protein levels were also measured in the SNpc and STR using Western blot (**C**,**D**) (n = 3), Scale bars, 100 µm. Values are mean ± SD; ^##^ *p* < 0.01, ^###^ *p* < 0.001 vs. control group; * *p* < 0.05, ** *p* < 0.01 and *** *p* < 0.001 vs. MPTP-treated group.

Microglia and astrocytes play key roles in neuroinflammation, which contributes to the progression of PD. To evaluate the anti-neuroinflammatory effects of OTPM, we used an MPTP-induced PD mouse model. Immunostaining for MAC-1 revealed a marked increase in activated microglia in the STR and SNpc of MPTP-treated mice compared to vehicle controls. Oral administration of OTPM (10 mg/kg/day for 7 days) reduced MAC-1 expression, as confirmed by both immunohistochemistry (Figure 6A) and immunoblot analysis (Figure 6C). GFAP immunostaining showed elevated astrocyte activation in MPTP-intoxicated mice, which was attenuated by OTPM treatment (Figure 6B,C). These effects were also reflected in immunoblot results, demonstrating that OTPM effectively restored GFAP expression in the striatum and SNpc (Figure 6D). The above results suggest that OTPM attenuates microglia and astrocyte activation and can prevent MPTP-induced overexpression of neuroinflammatory responses.

### 2.7. Improvement of Behavior by OTPM in the MPTP-Induced PD Mouse Model

**Figure 7:**
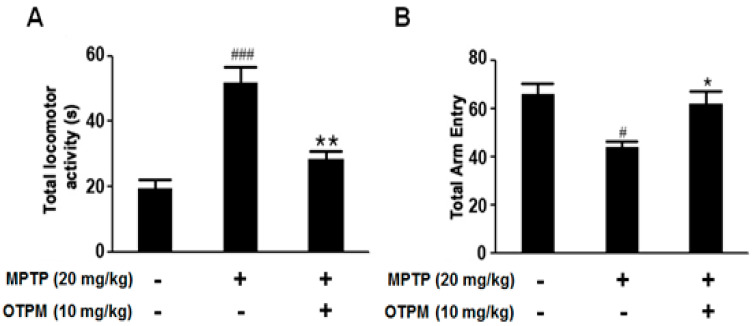
The effect of OTPM on behavioral deficits in MPTP-intoxicated PD mice. C57/BL6 mice were dosed with OTPM at 10 mg/kg, p.o., for 7 days before MPTP administration (20 mg/kg, i.p., 4 injections/day at 2 h intervals). Total Locomotor Activity (**A**) was recorded with a cut-off limit of 60 s in the Pole test. Total arm entry was evaluated using the Y-maze (**B**). Values are mean ± SD (n = 12); ^#^ *p* < 0.05, ^###^ *p* < 0.001 vs. control group; * *p* < 0.05 and ** *p* < 0.01 vs. MPTP-treated group.

A murine MPTP-induced PD model was used to evaluate the neuroprotective effects of OTPM. Mice received OTPM (10 mg/kg) for 7 days to investigate its potential mechanism of action. Bradykinesia was assessed using the pole test, which showed that MPTP significantly increased the time to reach the platform (total locomotor activity, *p* < 0.001 vs. vehicle). OTPM treatment significantly reduced this time (** *p* < 0.01 vs. MPTP) (Figure 7A). Spontaneous alternation behavior was assessed with the Y-maze test. MPTP reduced total arm entries (# *p* < 0.05 vs. vehicle), whereas OTPM treatment restored arm entry numbers (* *p* < 0.05) (Figure 7B). These results indicate that OTPM effectively alleviates motor dysfunction in PD mice.

## 3. Discussion

Neuroinflammation is a common pathological feature in the onset and progression of many NDDs, including PD. Although PD is mainly characterized by the degeneration or dysfunction of dopaminergic neurons, leading to neurochemical imbalances and impaired motor and non-motor functions, growing evidence indicates that neuroinflammation also plays a key role in disease progression. During acute neuroinflammation, inflammatory mediators act protectively to repair damaged neurons and glial cells. However, when inflammation becomes chronic, it contributes to neuronal injury and degeneration. The transcription factor NF-κB is a central player in PD pathogenesis, being involved in both α-synuclein aggregation and the regulation of inflammatory and cytokine signaling pathways. Studies suggest that α-synuclein can activate NF-κB in microglia, leading to increased cytokine production, whereas inhibition of NF-κB reduces microglial-induced neurotoxicity. Therefore, targeting NF-κB pathways and related inflammatory signaling may help suppress neuroinflammation and slow NDD progression. Most existing therapeutic drugs primarily target symptomatic relief and have notable limitations. Therefore, developing alternative therapeutic strategies is essential for effective PD management. OTPM, a bioactive derivative of fluoxetine, has demonstrated promising antidepressant properties by selectively modulating 5-hydroxytryptamine (5-HT). Fluoxetine is referenced in this study because of its well-known function as a selective serotonin reuptake inhibitor and its established relevance to neuroprotection. However, no direct comparative evaluation between OTPM and fluoxetine was performed. As a result, the present study cannot determine whether OTPM shares, exceeds, or differs from fluoxetine in its pharmacological effects. Instead, this work focuses on assessing the neuroprotective and anti-inflammatory actions of OTPM in LPS-stimulated BV-2 microglial cells and MPTP-induced PD mouse models.

LPS, a bacterial endotoxin, is widely used to induce microglial activation and model neuroinflammation [22]. The LPS-induced experimental model provides valuable insight into the role of inflammation in NDD progression [23,24]. Upon binding to cell surface receptors, LPS activates intracellular signaling cascades that stimulate the release of inflammatory mediators, including NO. Excessive NO production is cytotoxic and contributes to neuronal damage [25,26]. In this study, we evaluated the effect of OTPM on LPS-stimulated BV-2 microglial cells to identify potential therapeutic targets for PD and related inflammatory disorders. Our findings showed that OTPM pretreatment significantly reduced NO production and improved cell viability in a dose-dependent manner, consistent with previous reports demonstrating the anti-inflammatory effects of OTPM and its structurally similar fluoxetine derivative in BV-2 microglial cells [20]. NO plays a crucial role in the inflammatory response as a major cytotoxic mediator produced by activated microglia [27]. Its production is regulated by iNOS, which is normally expressed at low levels but becomes upregulated upon microglial activation, contributing to neuronal cell death [28]. The transcription of the iNOS gene is further stimulated by pro-inflammatory cytokines such as TNF-α, IL-1β, and IL-6, promoting chronic inflammation [29]. During neuroinflammation, TNF-α, IL-6, and IL-1β are released and trigger the expression of chemokines, thereby increasing neuronal damage [30,31]. In PD rat models, fluoxetine has been shown to inhibit the expression of TNF-α, IL-1β, and iNOS following LPS administration [32]. Similarly, our findings demonstrate that OTPM suppresses the production of TNF-α, IL-1β, and IL-6 by downregulating their mRNA expression. These results align with previous reports indicating that fluoxetine and its derivatives reduce TNF-α, IL-1β, and NO production and decrease the expression of related inflammatory mRNAs in activated microglia and macrophages [32,33]. It was previously demonstrated that both iNOS and COX-2 are overexpressed in activated microglia, contributing to neurodegeneration. In LPS-stimulated BV-2 microglial cells, NO production increases in a concentration-dependent manner, triggering the release of pro-inflammatory mediators such as COX-2 and iNOS, thereby amplifying neuroinflammatory responses [34,35]. Treatment of BV-2 cells with LPS has been shown to significantly elevate COX-2 and iNOS expression at both the protein and mRNA levels, promoting inflammation [36,37]. Specifically, exposure to 0.1–1.0 μg/mL LPS for various durations increased COX-2 and iNOS expression by approximately 1.5- to 80-fold compared with untreated controls [38,39]. Consistent with previous findings, the current study also observed that inflammatory genes were simultaneously upregulated following LPS stimulation [40,41]. However, treatment with OTPM effectively inhibited the expression of inflammatory mediators, including iNOS and COX-2, in BV-2 microglial cells. This inhibition corresponded with a reduction in NO production and microglial activation. Since activated microglia drive inflammatory responses and exacerbate neuronal cell death, OTPM’s suppressive effects highlight its potential as a neuroprotective and anti-inflammatory agent.

In microglial cells, LPS binds to Toll-like receptor 4 (TLR4) on the cell membrane, activating several intracellular signaling pathways, including the NF-κB pathway via the MyD88–IRAK–TRAF6–TAK1 complex [42]. This activation stimulates the IκB kinase (IKK) complex, leading to phosphorylation and subsequent degradation of IκBα. As a result, NF-κB is released from the NF-κB/IκBα complex and translocates to the nucleus, where it functions as a transcription factor to promote the expression of pro-inflammatory mediators such as NO, IL-6, TNF-α, and reactive oxygen species [43,44]. In the present study, we examined whether OTPM modulates LPS-induced NF-κB activation in BV-2 microglial cells. OTPM treatment effectively suppressed the LPS-induced phosphorylation and degradation of IκBα, thereby preventing NF-κB activation and nuclear translocation. These findings indicate that OTPM inhibits NF-κB signaling by stabilizing IκBα and blocking its phosphorylation and degradation. Since the IKKβ subunit of the IKK complex mediates IκBα phosphorylation and degradation in response to inflammatory stimuli [45,46], OTPM’s effect may involve interference with IKK activity, ultimately reducing the inflammatory response. Our findings align with previous studies by Song et al. [47], Koh et al. [48], Tian et al. [49], and Yang et al. [50], which reported that fluoxetine and its derivatives possess anti-inflammatory properties. These studies demonstrated that such compounds suppress NF-κB activation and reduce inflammatory responses in various models of neurotoxicity.

C57BL/6 mice exposed to the Parkinsonian neurotoxin MPTP exhibited abnormal dopaminergic (DA) transmission and motor dysfunction. MPTP-induced neuronal DA depletion led to marked reductions in motor activity [51]. The pole test and Y-maze task, a validated behavioral task for motor and cognitive function in rodents, confirmed the presence of these deficits [52]. MPTP-treated mice showed impaired motor coordination and cognitive performance, with significantly increased total locomotor activity and decreased total arm entries compared to saline-treated controls. Pretreatment with OTPM prior to MPTP administration markedly improved both motor and cognitive performance in the pole and Y-maze tests, suggesting a protective effect. MPTP is known to activate microglia and astrocytes in the substantia nigra pars compacta (SNpc) and striatum (STR), processes central to the pathology of PD and other NDDs. This glial activation is typically accompanied by increased expression of MAC-1 (a microglial marker) and GFAP (an astrocytic marker) [53]. Under neurodegenerative conditions, reactive glial cells release inflammatory mediators that contribute to neuronal damage [54]. Elevated MAC-1 and GFAP expression has been reported in both PD patients and MPTP models, indicating that gliosis-related inflammation contributes to dopaminergic neuronal loss [55,56,57,58]. In the present study, OTPM treatment significantly reduced the MPTP-induced upregulation of GFAP and MAC-1 in the SNpc and STR, indicating inhibition of microglial and astrocytic activation. These findings suggest that OTPM exerts neuroprotective effects by suppressing glial activation and associated neuroinflammation, thereby alleviating motor symptoms and dopaminergic neuronal damage in PD.

## 4. Materials and Methods

### 4.1. Reagents

LPS, 1-methyl-4-phenyl-1,2,3,6-tetrahydropyridine (MPTP), bovine serum albumin (BSA), dimethyl sulfoxide (DMSO), and 3-(4,5-dimethylthiazol-2-yl)-2,5-diphenyltetrazolium bromide (MTT) were purchased from Sigma-Aldrich (St. Louis, MO, USA). 10× RIPA buffer was obtained from Millipore (Milford, MA, USA), and protease and phosphatase inhibitor cocktail tablets were supplied by Roche (Indianapolis, IN, USA). Dulbecco’s modified Eagle’s medium (DMEM), fetal bovine serum (FBS), phosphate-buffered saline (PBS), and other cell culture reagents were obtained from Gibco-BRL Technologies (Carlsbad, CA, USA). All other analytical-grade chemicals were purchased from Sigma-Aldrich (St. Louis, MO, USA).

### 4.2. Synthesis of OTPM

Paraformaldehyde (415 mg, 13.8 mmol) and *N*,*N*-dimethylamine hydrochloride (1.13 g, 13.8 mmol) were added dropwise to a solution of 2-(trifluoromethyl)acetophenone (2.00 g, 10.6 mmol) in acetonitrile (35 mL) containing sulfuric acid (0.1 mL). The mixture was stirred under reflux for 9 h, after which acetone (35 mL) was added. The resulting solution was cooled to −10 °C and stirred for 20 min. The precipitate was filtered to yield *N*,*N*-dimethyl-3-oxo-3-(2-(trifluoromethyl)phenyl)propan-1-aminium chloride (compound **2**) as a white solid (1.90 g, 59.2%). Compound **2** (500 mg, 1.78 mmol) was dissolved in morpholine (7.74 g, 88.9 mmol) and heated to reflux for 30 min with stirring. After completion, the reaction mixture was concentrated under reduced pressure and extracted with diethyl ether. The organic layer was washed with brine, dried over anhydrous Na_2_SO_4_, and concentrated in vacuo to obtain the crude product. Purification by silica gel column chromatography (dichloromethane:methanol = 9:1) yielded the free amine product. To obtain the HCl salt, the free amine was dissolved in diethyl ether, and HCl solution (2.0 M in diethyl ether, 30 mL) was added at −10 °C. The resulting precipitation was filtered and washed with ether to afford OTPM as a white solid (240 mg, 41.7%). Mp > 168 °C (decomposed); infrared (KBr, cm^−1^) 1706; ^1^H nuclear magnetic resonance (NMR) (400 MHz, DMSO-*d*_6_) δ 7.94 (d, 1H, *J* = 7.6 Hz, Ph-H), 7.87 (t, 2H, *J* = 8.0 Hz, Ph-H), 7.77 (t, 1H, *J* = 7.6 Hz, Ph-H), 3.94 (d, 2H, *J* = 12.0 Hz, CH_2_), 3.82 (t, 2H, *J* = 12.0 Hz, CH_2_), 3.67 (t, 2H, *J* = 7.6 Hz, CH_2_), 3.45 (m, 4H, CH_2_), 3.10 (t, 2H, *J* = 9.2 Hz, CH_2_). ^13^C NMR (100 MHz, DMSO-*d*_6_) δ 199.67, 137.97, 137.01, 132.50, 131.25, 128.02, 126.70, 63.11, 51.11, 50.42, 36.36. ESI-MS: [M+H]^+^ 288.10 [20].

### 4.3. Cell Culture and Treatment

BV-2 microglial cells were obtained as previously described [38]. The cells were maintained in DMEM supplemented with 5% FBS and 50 µg/mL penicillin–streptomycin in a humidified incubator at 37 °C with 5% CO_2_ and 95% O_2_. For experiments, cells were seeded at a density of 5 × 10^4^ cells/mL, pretreated with various concentrations of OTPM (1, 5, and 25 µM) for 1 h, and then stimulated with LPS at 100 ng/mL for 24 h.

### 4.4. Animals and Treatment

Six-week-old male C57BL/6 mice were obtained from Samtako Bio Korea (Osan, Republic of Korea) and acclimatized before experimentation. Mice aged approximately eight weeks and weighing 25–28 g were used in this study. All experimental procedures followed the Principles of Laboratory Animal Care (NIH) and the Guidelines for Animal Experiments at Konkuk University (Approval NO: KU13167). The animals were housed under controlled conditions (23 ± 1 °C, 50% ± 5% humidity) with free access to food and water. The light cycle was maintained from 08:00 to 20:00. A total of 36 mice were randomly assigned to three groups (n = 12 per group): Vehicle control, MPTP (20 mg/kg, four injections/day at 2 h intervals), and OTPM (10 mg/kg, orally) + MPTP (20 mg/kg). OTPM was freshly dissolved in triple-distilled water before administration and given orally for seven consecutive days prior to MPTP injection. The vehicle group received an equivalent volume of triple-distilled water following the same schedule. Behavioral assessments were conducted 36 h after the final MPTP injection. The following morning, all mice were sacrificed for further experimental analyses. After acquiring the SNpc and striatum from the brain, we divided it into four sets for experimental analysis. The first and second sets of mice were used to analyze various biomarkers of neuroprotective and anti-neuroinflammatory effects by Western blot and RT-PCR (Supplementary Figure S1). The third set of mice was used to assess MAC-1 and GFAP expression in microglia and astrocytes in the SNpc and striatum by immunohistochemistry. The fourth set of mice we used to evaluate the antioxidant activity ). In this study, no mortality or unexpected adverse effects were observed during MPTP administration or throughout the behavioral testing period. All mice completed the experimental procedures without complications, and no animals were excluded from the analysis.

### 4.5. Cell Viability and NO Assays

BV-2 microglial cells were seeded and treated with various concentrations of OTPM (1, 5, and 25 µM). After 24 h of incubation with LPS (100 ng/mL), MTT solution (0.5 mg/mL) was added to each well. The cells were then incubated for 2 h at 37 °C in a 5% CO_2_ atmosphere. Following incubation, the supernatant was removed, and the formazan crystals formed in viable cells were dissolved in DMSO. Absorbance was measured at 540 nm using a microplate reader (Tecan Trading AG, Basel, Switzerland). For NO measurement, BV-2 microglial cells (5 × 10^4^ cells/mL) were treated with LPS (100 ng/mL) in the presence or absence of OTPM (1, 5, and 25 µM) for 24 h. The culture supernatants were collected, and NO levels were quantified using the Griess reagent. Absorbance was recorded at 540 nm with a microplate reader. All experiments were performed in triplicate.

### 4.6. Total RNA Extraction and Reverse Transcription Polymerase Chain Reaction (RT-PCR)

Total RNA was isolated from BV-2 microglial cells using Trizol reagent (Invitrogen Life Technologies, Carlsbad, CA, USA) following the manufacturer’s protocol. First-strand cDNA was synthesized from 2.5 μg of total RNA using the ReverTra Ace-α kit (Toyobo, Osaka, Japan). PCR amplification was carried out with specific primers (Bioneer, Daejeon, Republic of Korea) as described previously [25]. The primer sequences are listed in Supplementary Table S1. PCR products were separated on a 1.2% agarose gel and visualized using ethidium bromide staining. The gels were photographed, and band intensities were quantified using ImageJ Version 1.53 software (NIH, Bethesda, MD, USA). Gene expression levels were normalized to GAPDH.

### 4.7. Western Blot Analysis

Brain tissues were homogenized and lysed in 1× RIPA buffer containing protease and phosphatase inhibitor cocktails. The lysates were centrifuged at 13,000 rpm for 15 min at 4 °C, and the supernatants were collected for further analysis. For cell studies, BV-2 microglial cells were seeded in six-well plates at a density of 5 × 10^4^ cells/well and treated with OTPM (1, 5, and 25 µM) for 6 h, either alone or in combination with LPS (100 ng/mL). After treatment, cell lysates were prepared using 1× RIPA buffer and centrifuged at 13,000 rpm for 15 min at 4 °C. Protein concentrations were determined using the DC protein assay (Bio-Rad, Hercules, CA, USA). Equal amounts of protein (20–40 µg) were separated by 10% SDS–PAGE and transferred to polyvinylidene difluoride (PVDF) membranes (Millipore, Bedford, MA, USA). The membranes were blocked with 5% skim milk in PBS for 1 h at room temperature to prevent nonspecific binding, followed by incubation with primary antibodies overnight at 4 °C. After washing, membranes were incubated with the appropriate HRP-conjugated secondary antibodies for 2 h at room temperature. Protein bands were visualized using an enhanced chemiluminescence (ECL) detection system, and quantified with NIH ImageJ software (Version 1.53). All Western blot analyses were performed in triplicate.

### 4.8. Immunocytochemistry

BV-2 microglial cells (5 × 10^4^ cells/well) were cultured on sterile 12 mm coverslips in 24-well plates and treated with OTPM (25 µM) and LPS (100 ng/mL) to examine the intracellular localization of the NF-κB (p65) subunit. Immunocytochemistry was performed as previously described [23]. More than 50 cells per field were analyzed using a fluorescence microscope (Carl Zeiss Inc., Oberkochen, Germany).

### 4.9. Immunohistochemistry

After completing the behavioral experiments, groups of mice treated with MPTP or OTPM were anesthetized with sodium pentobarbital (50 mg/kg, i.p.) for immunohistochemical analysis. The brains were perfused with saline, followed by 4% paraformaldehyde in 0.1 M phosphate buffer (pH 7.4). After perfusion, brains were removed, post-fixed in the same fixative at 4 °C, and then dehydrated in 30% sucrose before embedding in tissue freezing medium (Leica, GmbH, Heidelberg, Germany). Frozen sections (30 µm) of the STR and SNpc were prepared for immunohistochemistry. All sections were incubated with primary antibodies: anti-MAC-1 rabbit antibody (1:250; AbD Serotec, Oxford, UK) and rabbit polyclonal anti-GFAP antibody (1:5000; Abcam, Cambridge, UK). The following day, sections were incubated for 60 min at room temperature with secondary antibodies: Alexa Fluor 594-labeled donkey anti-rabbit (1:500) and Alexa Fluor 488-labeled donkey anti-rabbit (1:5000; Molecular Probes Inc., Eugene, OR, USA). Sections were washed three times with 0.05% Tween-20 in PBS (5 min each), mounted with Vectashield (Vector Laboratories, Burlingame, CA, USA), and imaged using a fluorescence microscope (Carl Zeiss Inc., Oberkochen, Germany).

### 4.10. Behavioral Experiments: Pole Test and Y-Maze Test

Bradykinesia was evaluated using a modified pole test and Y-maze spontaneous alternation, as previously described [59,60]. For the pole test, mice were placed facing upward at the top of a rough-surfaced pole (8 mm diameter, 55 cm height), and the time taken to descend to the floor was recorded. Longer descent times indicate bradykinesia. Each mouse underwent five consecutive trials. In the Y-maze test, spontaneous alternation behavior was assessed during a single 8 min session. The maze consisted of three arms, each 40 cm long, 12 cm wide, and 30 cm high. Naive mice were placed at the end of one arm and allowed to explore freely. Arm entries were recorded by an observer blinded to the treatment groups, with an entry counted when the mouse’s hind paws were fully inside the arm. Alternation was defined as successive entries into all three arms in overlapping triplet sequences. All behavioral assessments were conducted by an experimenter blinded to the treatment groups. Animals were randomly assigned to each group using a random number generator to ensure unbiased allocation. Each experiment was performed in triplicate to confirm reproducibility.

### 4.11. Statistical Analysis

All data were analyzed using GraphPad Prism version 5.01 (GraphPad, Inc., La Jolla, CA, USA) and are presented as the mean ± standard deviation (SD) from at least three independent experiments. Statistical comparisons were performed using one-way analysis of variance (ANOVA) followed by Tukey’s multiple comparison test. A *p*-value of <0.05 was considered statistically significant.

## 5. Conclusions

The pathology of many NDDs is closely linked to heightened inflammatory responses within the CNS. Despite this, the development of effective pharmacological treatments remains challenging. Natural compounds and their derivatives have emerged as promising therapeutic candidates due to their efficacy and reduced adverse effects, aligning with the neuroinflammatory hypothesis of Parkinson’s disease (PD). In the present study, OTPM demonstrated significant anti-inflammatory activity in vitro. It inhibited nitric oxide (NO) production, reduced the release of pro-inflammatory cytokines (TNF-α, IL-1β, and IL-6), and suppressed iNOS and COX-2 expression in LPS-stimulated BV-2 microglial cells, without inducing cytotoxicity. These effects were primarily mediated through the inhibition of NF-κB signaling. In vivo, OTPM treatment mitigated microglial and astrocytic activation, thereby suppressing neuroinflammatory responses and preventing motor impairments in the MPTP-induced mouse model of PD. These findings indicate that OTPM exerts a neuroprotective effect by modulating glial activity and reducing neuroinflammation. Overall, this study provides experimental evidence supporting OTPM as a potential therapeutic candidate for PD and other neuroinflammatory disorders.

Given its demonstrated neuroprotective and anti-inflammatory effects, further investigations should focus on elucidating the detailed molecular mechanisms underlying its action, along with comprehensive pharmacokinetic, bioavailability, and safety evaluations in preclinical models to facilitate its progression toward clinical trials.

### Limitations

Although the present study demonstrates the neuroprotective potential of OTPM, it has some limitations. OTPM has been reported to exhibit antidepressant-like effects in previous behavioral studies; its specific molecular targets have not been identified. The reference to selective 5-HT modulation was based on indirect evidence from serotonin-related behavioral assays rather than receptor-binding data. The comparison to fluoxetine was intended to highlight a possible functional similarity in neuromodulation, not structural or mechanistic equivalence. Further studies are required to elucidate the precise molecular targets and receptor-level interactions of OTPM. Further, long-term neurotoxicity assessments were not performed, and dopaminergic neuron survival was not quantified using TH immunostaining. Future studies incorporating these evaluations will be necessary to further substantiate OTPM’s neuroprotective efficacy. In addition, pharmacological considerations, such as OTPM pharmacodynamic variability, its metabolic fate, and its ability to cross the blood–brain barrier (BBB), warrant further investigation. The extent to which active forms of OTPM reach the CNS remains unclear. Therefore, future studies should evaluate the in vivo pharmacokinetic properties of OTPM and explore delivery systems that enhance BBB penetration and metabolic stability. Future studies will evaluate the direct effects of OTPM on MAO-A and MAO-B activity to determine whether MAO inhibition contributes to its neuroprotective and anti-inflammatory effects. Finally, future research will include established reference drugs such as fluoxetine, selegiline, and L-DOPA to provide a more thorough comparison of OTPM’s therapeutic potential. Overall, the findings suggest that OTPM exerts neuroprotective and anti-inflammatory effects in LPS-stimulated BV-2 cells and MPTP-induced PD mice by modulating key pathways and inhibiting microglia–astrocyte activation. Further in vitro and in vivo studies are needed to clarify its mechanisms and therapeutic potential.

## Data Availability

The raw data supporting the conclusions of this article will be made available by the authors on request.

## Supplementary Materials

The following supporting information can be downloaded at https://www.mdpi.com/article/10.3390/ph18121799/s1, Table S1: Detailed forward and backward primer sequences used in this current study; Figure S1: Effect of OTPM on pro-inflammatory mediators and cytokines in MPTP-stimulated PD mice; File S1: Raw data of in vitro and in vivo studies.
