# Supplementary material for: Neuroprotective Effects of Fluoxetine Derivative 4-[3-Oxo-3-(2-trifluoromethyl-phenyl)-propyl]-morpholinium Chloride (OTPM) as a Potent Modulator of Motor Deficits and Neuroinflammatory Pathways in LPS-Induced BV-2 Microglial Cells and MPTP-Induced Parkinsonian Models
Source: Pharmaceuticals (Basel). 2025 Nov 26;18(12):1799. doi: 10.3390/ph18121799 (PMC12735652; doi:10.3390/ph18121799)
Supplement: Supplementary file 1 [file pharmaceuticals-18-01799-s001.zip › Figure S1. Inflammatory mediators and cytokines.pdf]

Supplementary Figure S1.

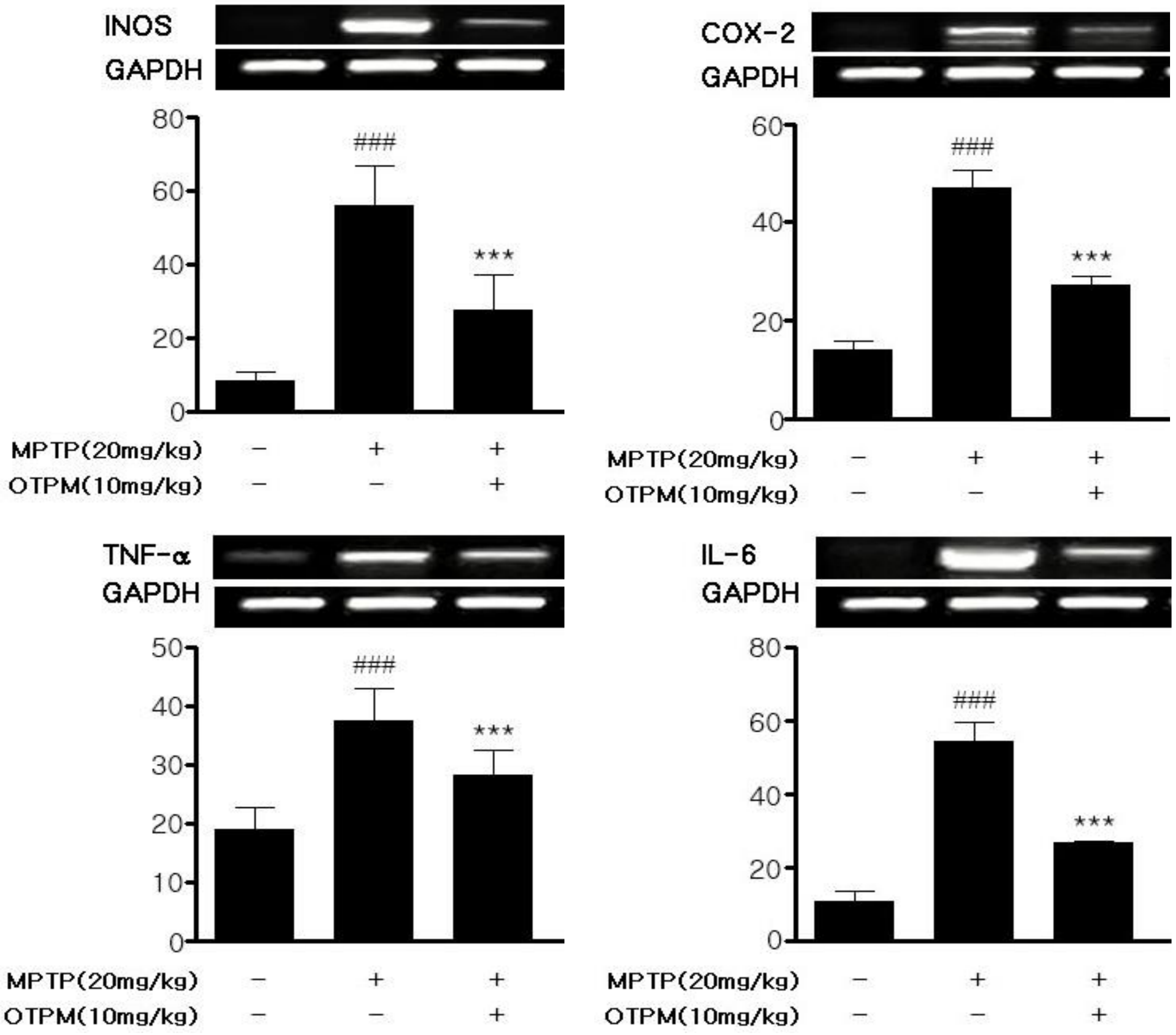

Supplementary Figure S1. Effect of OTPM on pro-inflammatory mediators and cytokines in MPTP-stimulated PD mice. The mRNA levels of iNOS, COX-2, TNF-α, and IL-6 were measured via RT-PCR. GAPDH served as an internal control. Data are expressed as mean ± SD (n=3). ###P < 0.001, vs. control group; ***P < 0.001, vs. MPTP-treated group, determined by one-way ANOVA.
