# Supplementary material for: Neuroprotective Effects of Fluoxetine Derivative 4-[3-Oxo-3-(2-trifluoromethyl-phenyl)-propyl]-morpholinium Chloride (OTPM) as a Potent Modulator of Motor Deficits and Neuroinflammatory Pathways in LPS-Induced BV-2 Microglial Cells and MPTP-Induced Parkinsonian Models
Source: Pharmaceuticals (Basel). 2025 Nov 26;18(12):1799. doi: 10.3390/ph18121799 (PMC12735652; doi:10.3390/ph18121799)
Supplement: Supplementary file 1 [file pharmaceuticals-18-01799-s001.zip › Table S1.pdf]

Table S1: Detailed forward and backward primer sequences used in this current study.

Gene		Sequence (5’→3’)	Accession No.	bp
iNOS	Forward	GAGGTACTCAGCGTGCTCCA	NM_010927	444
	Reverse	AGGGAGGAAAGGGAGAGAGG		
COX-2	Forward	TGAGTGGTAGCCAGCAAAGC	NM_011198	319
	Reverse	CTGCAGTCCAGGTTCAATGG		
IL-1β	Forward	CAAGGAGAACCAAGCAACGA	NM_008361	428
	Reverse	TTGGCCGAGGACTAAGGAGT		
IL-6	Forward	GGAGGCTTAATTACACATGTT	NM_031168	435
	Reverse	TGATTTCAAGATGAATTGGAT		
TNF-α	Forward	AGGGAGAGTGGTCAGGTTGC	NM_013693	392
	Reverse	CAGCCTGGTCACCAAATCAG		
